# A Novel Inflammation and Insulin Resistance Related Indicator to Predict the Survival of Patients With Cancer

**DOI:** 10.3389/fendo.2022.905266

**Published:** 2022-06-20

**Authors:** Guo-Tian Ruan, Hai-Lun Xie, He-Yang Zhang, Chen-An Liu, Yi-Zhong Ge, Qi Zhang, Zi-Wen Wang, Xi Zhang, Meng Tang, Meng-Meng Song, Xiao-Wei Zhang, Ming Yang, Yong-Bing Chen, Kai-Ying Yu, Li Deng, Yi-Zhen Gong, Wen Hu, Kun-Hua Wang, Ming-Hua Cong, Han-Ping Shi

**Affiliations:** ^1^ Department of Gastrointestinal Surgery, Beijing Shijitan Hospital, Capital Medical University, Beijing, China; ^2^ Department of Clinical Nutrition, Beijing Shijitan Hospital, Capital Medical University, Beijing, China; ^3^ Key Laboratory of Cancer Food for Special Medical Purposes (FSMP) for State Market Regulation, Beijing, China; ^4^ Department of Gastrointestinal Surgery, Guangxi Medical University Cancer Hospital, Nanning, China; ^5^ Guangxi Clinical Research Center for Colorectal Cancer, Nanning, China; ^6^ Clinical Nutrition Department, Sichuan University West China Hospital, Chengdu, China; ^7^ Yunnan University, Kunming, China; ^8^ General Surgery Clinical Medical Center of Yunnan Province, Kunming, China; ^9^ Comprehensive Oncology Department, National Cancer Center/Cancer Hospital, Chinese Academy of Medical Sciences and Peking Union Medical College, Beijing, China

**Keywords:** systemic inflammation, CRP, insulin resistance, TyG, overall survival

## Abstract

**Background:**

Systemic inflammation and insulin resistance (IR) are closely related in patients with cancer. However, there is no relevant indicator that combines inflammation and IR to predict patient prognosis. Therefore, this study aimed to develop and validate a novel inflammation- and IR-related marker in patients with cancer.

**Methods:**

The total cohort of this study included 5221 patients with cancer, and the training and validation cohorts were randomized in a 7:3 ratio. C-reactive protein (CRP) and fasting triglyceride glucose (TyG) were used to reflect patients’ inflammation and IR status, respectively. The CRP-TyG index (CTI) was composed of CRP and TyG. The concordance (C)-index, receiver operator characteristic (ROC) curve, and calibration curve reflected the prognostic predictive power of CTI. Univariate and multivariate survival analyses predicted the prognostic value of CTI in patients with cancer.

**Results:**

The C-indices of CTI in patients with cancer were 0.636, 0.617, and 0.631 in the total, training, and validation cohorts, respectively. The 1-, 3-, and 5-year ROC and calibration curves showed that CTI had a good predictive ability of survival in patients with cancer. Meanwhile, patients with high CTI had a worse prognosis compared to patients with low CTI (total cohort: hazard ratio [HR] = 1.46, 95% confidence interval [95% CI] = 1.33–1.59; training cohort: HR = 1.36, 95% CI = 1.22–1.52; validation cohort: HR = 1.73, 95% CI = 1.47–2.04].

**Conclusion:**

The CTI is a useful prognostic indicator of poor prognosis and a promising tool for treatment strategy decision-making in patients with cancer.

## Introduction

The American Cancer Society’s assessment of cancer incidence data in the United States shows that there will be 1,918,030 new cancer cases and 609,360 cancer deaths in 2022 (1). China’s population accounts for one-fifth of the world’s population. With the rapid socioeconomic development and population aging, China, similar to many other developing countries, is facing unprecedented challenges in cancer prevention and control (2). Due to its large population, the current cancer burden in China significantly affects the global scale of cancer (3).

Inflammation and insulin resistance (IR) play important roles in cancer (4). Cancer is an inflammatory disease, and systemic inflammation is a hallmark of patients with cancer (5). Inflammation is involved in the occurrence and progression of cancer, and systemic inflammatory state is considered to be the seventh hallmark of cancer formation through host-tumor interactions (6, 7). The inflammatory state is the main driving force behind the metabolic alterations in patients with cancer (8). The origin of inflammation is multifaceted: on the one hand, tumor cells may release cytokines and other inflammatory mediators; on the other hand, activated immune cells release cytokines and chemokines (9). The production of acute-phase proteins, such as C-reactive protein (CRP) and fibrinogen, is considered an accurate measure of systemic inflammation and pro-inflammatory cytokine activity (10). Elevated CRP levels demonstrate a systemic inflammatory response (8). A previous study showed that interleukin-6 (IL-6) inhibits hepatic albumin production in patients with pancreatic cancer and is positively correlated with serum CRP levels (11). CRP is a strong prognostic indicator of tumor progression, survival, and symptom burden in multiple cancers (12).

Insulin, an anabolic hormone, coordinates the oxidation or storage of glucose in the body. Insulin sensitivity is coordinated by the uptake of glucose by insulin-sensitive cells in muscle, fat, and the liver and the removal of glucose from the circulation when glucose is elevated (13). IR is generally defined as impaired tissue glucose uptake and inhibition of hepatic glucose production (14). IR is a key component of metabolic syndrome, morbidity and mortality are increased in IR patients, mainly due to cardiovascular disease and type 2 diabetes (T2D) (15, 16). In addition, many epidemiological studies have consistently shown that patients with IR are associated with an increased risk of cancers (including that of the breast, liver, colorectum, and pancreas) (17). IR in patients with cancer is characterized by increased hepatic glucose production and gluconeogenesis, and different from T2D, normal fasting glucose is associated with high, normal, or low levels of insulin (13). This may be due to the redistribution of glucose within tumor cells to supply energy requirements (18). The increased glucose demand of cancer cells can lead to hypoglycemia, leading to increases in compensatory hormonal signaling, growth hormone, epinephrine, or glucagon. For instance, activation of IR can stimulate the PI3K/Akt/mTOR pathway and the MAP/ERK kinase pathway, ultimately leading to cell proliferation and migration and inhibiting apoptosis (19). In addition, tumor by-products may lead to IR. Tumor by-products in patients with lung cancer, including corticotropin and corticotropin-releasing factor, may contribute to abnormal glucose metabolism (20). Chronic IR is found in malignant tumors and presumed to contribute to cancer cachexia due to chronic exposure to proinflammatory cytokines, tumor necrosis factor (TNF)-α, IL-6, and insulin growth factor-binding protein, leading to IR (21, 22). A study by Xia et al. has reported that inflammatory responses play an important role in the development of IR through the adaptive immune system (23). In a study of moderate weight loss in 10 men with non-small cell lung cancer, IR was associated with 26% of protein anabolism, which was associated with CRP, but not with weight loss (14).

Currently, the logarithmic product of fasting triglyceride and glucose levels (labeled as the TyG index) is considered a simple measure of IR and has been reported in many tumor-related studies (24–26). Because of the close association between inflammation and IR, their interaction might help predict mortality in patients with cancer. However, to the best of our knowledge, there is no prognostic indicator related to inflammation and IR to predict the survival of patients with cancer. Hence, this study aimed to develop and validate a new inflammatory IR indicator (composed of CRP and TyG) to predict the survival of patients with cancer.

## Materials and Methods

### Study Population

This was a prospective, cross-sectional, multicenter cohort study-”INSCOC” (Investigation on Nutrition Status and its Clinical Outcome of Common Cancers) (27), which collected the clinical information from patients with cancer in China between 2013 and 2021. The INSCOC cohort prospectively collected data on nutrition and clinical outcomes in patients with cancer, and the cohort was followed up for 8 years. Inclusion criteria for this cohort included: 1. 18 years and older; 2. Pathologically diagnosed with cancer; 3. Clear consciousness and no communication difficulties. There are no strict exclusion criteria. A total of 22783 patients with cancer were initially included, and after deletion of missing data [age (n=12), height (n=1795), albumin (n=35), globulin (n=151), C-reactive protein (n =13583), glucose (n=111), cholesterol(n=1749), neutrocyte(n=83), lymphocyte (n=8), and platelet (n=35)], we obtained 5221 patients with cancer with complete clinical information. A total of 2149 events occurred during the 8-year follow-up of 5221 cancer patients. Finally, a 7:3 randomization of 5221 patients resulted in a training cohort (3657) and a validation cohort (1564) (**Figure S1**).

This study was conducted in accordance with the Declaration of Helsinki and was ethically approved by the ethics committees of the various research centers. Moreover, patients provided informed consent for inclusion in this study.

### Clinical and Laboratory Evaluations

The data collected in this study mainly included demographic characteristics (age, sex, body mass index [BMI]), lifestyle habits (smoking, yes/no; alcohol consumption, yes/no), comorbidities (hypertension, yes/no; diabetes, yes/no; coronary heart disease, yes/no), tumor-related information (surgery, yes/no; radiotherapy, yes/no; chemotherapy, yes/no; tumor-node-metastasis (TNM) stage), Karnofsky Performance Status (KPS), nutritional information (Scored Patient-Generated Subjective Global Assessment [PGSGA]; nutritional intervention, yes/no), body measurements (triceps skin fold thickness [TSF]), and laboratory tests (total cholesterol [TC], blood glucose, CRP).

All questionnaires, body measurements, information collection, and laboratory sample collection were completed within 48 h of patient admission. Patient questionnaires were obtained by experienced physicians or nurses. BMI was calculated from the ratio of body weight (kg) to height squared (m^2^). The patient’s weight was measured when the patient was wearing a light hospital gown, and the height was obtained when the patient was wearing socks. Medical records were centrally reviewed by two study oncologists to confirm the the cancer diagnosis and staged the cancer based on the 8th edition of the American Joint Committee on Cancer staging manual. Fasting blood samples were taken from all participants at the start of the study. The calculation formula of the IR index TyG is as follows: Ln (TC [mg/dl] × FBG [mg/dl])/2. The inflammation-IR index constructed in this study was defined as C-reactive protein- triglyceride glucose index (CTI), and the calculation formula was 0.412* Ln (CRP) + TyG.

### Outcome Assessment

The primary endpoint of this study was overall survival (OS) in patients with cancer. OS was defined as the time from the patient’s initial diagnosis of cancer to the patient’s death or last follow-up. All patient follow-up information was obtained by dedicated and experienced follow-up department personnel. The patient’s follow-up information was obtained through the patient’s outpatient follow-up record information and patient readmission record, or by telephone consultation.

### Statistical Analyses

Patients were randomized into the training and validation cohorts in a 7:3 ratio by R software. At baseline, continuous variables are presented as mean ± standard deviation(SD)or median ± interquartile range, whereas categorical variables are presented as percentages (%). Comparisons between the two groups were performed using Student’s t-test for continuous data (If the data met the normal distribution after using the Shapiro-Wilk test, if not, used the Wilcoxon test.) and the χ^2^ test for categorical data. Statistical correlations between variables were calculated using Pearson correlation coefficients, when the correlation coefficients were greater than 3 or less than -3 and satisfied *P*<0.05, it was considered a significant correlation. The optimal cut-off value for CTI was calculated using the maximum selected rank statistic, which was 18.67 (**Figure S2**). For time-event analyses, survival estimates were calculated using the Kaplan–Meier method and compared between groups using the log-rank test. Cox proportional hazards models were used to estimate hazard ratios (HRs) and 95% confidence interval (95% CI) for cancer death. All Cox proportional hazards models were tested for Proportional hazards after Schoenfeld’s test. Moreover, the prognostic performance of CTI was analyzed in the training and validation cohorts. In the multivariate survival analysis, we used different adjusted models, namely, model 0 (unadjusted), model 1 (adjusted for age, sex, BMI,and TNM stage), model 2 (adjusted for age, sex, TNM stage, BMI, tumor types, KPS, surgery, chemotherapy, radiotherapy, smoking, alcohol consumption, nutritional intervention, hypertension, coronary heart disease, and diabetes), and model 3 (adjusted for age, sex, TNM stage, BMI, tumor types, KPS, surgery, chemotherapy, radiotherapy, smoking, alcohol consumption, nutritional intervention, hypertension, coronary heart disease, diabetes, and TSF). All analyses were performed with R software version 4.0.3 using the software packages “timeROC, version 0.4” “rms, version 6.2-0” “survminer, version 0.4.9” “caret, version 6.0-9.0” and “survival, 3.2-11” and a two-sided *P* value <0.05 was considered statistically significant. *P*-values for interactions were generated using the interaction term in a Cox multivariate model.

## Results

### Baseline Characteristics

Clinicopathological characteristics of patients in the total, training, and validation cohorts are presented in **Table 1**. In the total, training, and validation cohorts, the mean ages of the patients were 59.41 ± 11.15, 59.38 ± 11.18, and 59.49 ± 11.07 years, respectively. Moreover, 3061 (58.6%), 2148 (58.7%), and 913 (58.4%) participants were male in the total, training, and validation cohorts, respectively. In the total, training, and validation cohorts, grouped according to the level of CTI, compared with patients with low CTI, patients with cancer with high CTI were older, comprised more male patients, and had lower KPS and higher PGSGA score (all *P* < 0.05) (**Table 2**).

**Table 1:** Baseline characteristics of the study population.

Characteristics	Total cohort	Training cohort	Validation cohort	*P*-value
	(n = 5221)	(n = 3657)	(n = 1564)	
Age (mean (SD))	59.41 (11.15)	59.38 (11.18)	59.49 (11.07)	0.751
Age (%)				0.663
<65	3454 (66.2)	2412 (66.0)	1042 (66.6)	
≥65	1767 (33.8)	1245 (34.0)	522 (33.4)	
Gender (%)				0.832
Male	3061 (58.6)	2148 (58.7)	913 (58.4)	
Female	2160 (41.4)	1509 (41.3)	651 (41.6)	
BMI (mean (SD))	22.57 (3.51)	22.54 (3.51)	22.65 (3.52)	0.298
BMI, kg/m^2^ (%)				
<18.5	616 (11.8)	442 (12.1)	174 (11.1)	0.369
18.5-23.9	2899 (55.5)	2034 (55.6)	865 (55.3)	
24-27.9	1162 (22.3)	816 (22.3)	346 (22.1)	
≥28	544 (10.4)	365 (10.0)	179 (11.4)	
Smoking, yes (%)	2431 (46.6)	1688 (46.2)	743 (47.5)	0.387
Alcohol, yes (%)	1167 (22.4)	823 (22.5)	344 (22.0)	0.712
Diabetes, yes (%)	530 (10.2)	381 (10.4)	149 (9.5)	0.354
Hypertension, yes (%)	1085 (20.8)	742 (20.3)	343 (21.9)	0.193
Coronary heart disease, yes (%)	268 (5.1)	175 (4.8)	93 (5.9)	0.094
Tumor types (%)				0.617
Lung cancer	1776 (34.0)	1226 (33.5)	550 (35.2)	
Esophageal cancer	313 (6.0)	234 (6.4)	79 (5.1)	
Gastric cancer	766 (14.7)	527 (14.4)	239 (15.3)	
Colorectal cancer	917 (17.6)	640 (17.5)	277 (17.7)	
Other digestive cancers	394 (7.5)	275 (7.5)	119 (7.6)	
Breast cancer	464 (8.9)	330 (9.0)	134 (8.6)	
Female reproductive cancer	177 (3.4)	130 (3.6)	47 (3.0)	
Urological cancer	132 (2.5)	90 (2.5)	42 (2.7)	
Nasopharyngeal cancer	126 (2.4)	92 (2.5)	34 (2.2)	
Other cancer	156 (3.0)	113 (3.1)	43 (2.7)	
TNM stage (%)				
I	441 (8.4)	320 (8.8)	121 (7.7)	0.640
II	943 (18.1)	658 (18.0)	285 (18.2)	
III	1413 (27.1)	980 (26.8)	433 (27.7)	
IV	2424 (46.4)	1699 (46.5)	725 (46.4)	
Surgery, yes (%)	2596 (49.7)	1827 (50.0)	769 (49.2)	0.622
Radiotherapy, yes (%)	581 (11.1)	404 (11.0)	177 (11.3)	0.813
Chemotherapy, yes (%)	3312 (63.4)	2317 (63.4)	995 (63.6)	0.882
KPS (mean (SD))	85.40 (12.35)	85.35 (12.56)	85.50 (11.86)	0.698
KPS (%)				
≥70	4914 (94.1)	3430 (93.8)	1484 (94.9)	0.141
<70	307 (5.9)	227 (6.2)	80 (5.1)	
PGSGA (mean (SD))	6.01 (4.76)	6.03 (4.76)	5.97 (4.74)	0.698
Nutritional intervention, yes (%)	1000 (19.2)	722 (19.7)	278 (17.8)	0.106
TSF, mm (mean (SD))	16.21 (8.85)	16.16 (8.81)	16.33 (8.96)	0.536
Tch, mmol/L (mean (SD))	4.60 (1.11)	4.59 (1.10)	4.62 (1.14)	0.416
Blood glucose, mmol/L (mean (SD))	5.78 (1.76)	5.78 (1.74)	5.77 (1.81)	0.779
CRP, mg/L (median (IQR))	3.71 (13.20)	3.71 (13.70)	3.70 (12.24)	0.391
TyG (mean (SD))	3.88 (0.29)	3.88 (0.29)	3.87 (0.29)	0.533
CTI (mean (SD))	4.57 (0.74)	4.57 (0.75)	4.57 (0.72)	0.999

SD, standard deviation; IQR, interquartile range; BMI, body mass index; KPS, karnofsky performance status; PGSGA, patient-generated subjective global assessment; Tch, total cholesterol; CRP, C-reactive protein; TyG, triglyceride glucose; CTI, C-reactive protein-triglyceride glucose index; TSF, triceps skin fold.

**Table 2:** Baseline characteristics stratified by CTI.

Variables	Total cohort			Training cohort			Validation cohort		
	Low CTI	High CTI	*P*-value	Low CTI	High CTI	*P*-value	Low CTI	High CTI	*P*-value
	(n = 3311)	(n = 1910)		(n = 2298)	(n = 1359)		(n = 1013)	(n = 551)	
Age, years (mean (SD))	58.69 (11.26)	60.68 (10.85)	<0.001	58.63 (11.37)	60.66 (10.75)	<0.001	58.82 (11.01)	60.72 (11.08)	0.001
Age, years (%)									
<65	2267 (68.5)	1187 (62.1)	<0.001	1572 (68.4)	840 (61.8)	<0.001	695 (68.6)	347 (63.0)	0.028
≥65	1044 (31.5)	723 (37.9)		726 (31.6)	519 (38.2)		318 (31.4)	204 (37.0)	
Gender (%)									
Male	1849 (55.8)	1212 (63.5)	<0.001	1292 (56.2)	856 (63.0)	<0.001	557 (55.0)	356 (64.6)	<0.001
Female	1462 (44.2)	698 (36.5)		1006 (43.8)	503 (37.0)		456 (45.0)	195 (35.4)	
BMI (mean (SD))	22.53 (3.43)	22.64 (3.64)	0.266	22.56 (3.43)	22.50 (3.62)	0.581	22.46 (3.42)	23.00 (3.67)	0.003
BMI, kg/m^2^ (%)			0.261			0.257			0.048
<18.5	384 (11.6)	232 (12.1)		261 (11.4)	181 (13.3)		123 (12.1)	51 (9.3)	
18.5-23.9	1862 (56.2)	1037 (54.3)		1292 (56.2)	742 (54.6)		570 (56.3)	295 (53.5)	
24-27.9	739 (22.3)	423 (22.1)		522 (22.7)	294 (21.6)		217 (21.4)	129 (23.4)	
≥28	326 (9.8)	218 (11.4)		223 (9.7)	142 (10.4)		103 (10.2)	76 (13.8)	
Smoking, yes (%)	1419 (42.9)	1012 (53.0)	<0.001	968 (42.1)	720 (53.0)	<0.001	451 (44.5)	292 (53.0)	0.002
Alcohol, yes (%)	690 (20.8)	477 (25.0)	<0.001	482 (21.0)	341 (25.1)	0.005	208 (20.5)	136 (24.7)	0.067
Diabetes, yes (%)	249 (7.5)	281 (14.7)	<0.001	181 (7.9)	200 (14.7)	<0.001	68 (6.7)	81 (14.7)	<0.001
Hypertension, yes (%)	591 (17.8)	494 (25.9)	<0.001	399 (17.4)	343 (25.2)	<0.001	192 (19.0)	151 (27.4)	<0.001
Coronary heart disease, yes (%)	162 (4.9)	106 (5.5)	0.670	105 (4.6)	70 (5.2)	0.474	57 (5.6)	36 (6.5)	0.540
Tumor types (%)			<0.001			<0.001			<0.001
Lung cancer	1014 (30.6)	762 (39.9)		693 (30.2)	533 (39.2)		321 (31.7)	229 (41.6)	
Esophageal cancer	200 (6.0)	113 (5.9)		149 (6.5)	85 (6.3)		51 (5.0)	28 (5.1)	
Gastric cancer	546 (16.5)	220 (11.5)		371 (16.1)	156 (11.5)		175 (17.3)	64 (11.6)	
Colorectal cancer	618 (18.7)	299 (15.7)		425 (18.5)	215 (15.8)		193 (19.1)	84 (15.2)	
Other digestive cancers	229 (6.9)	165 (8.6)		158 (6.9)	117 (8.6)		71 (7.0)	48 (8.7)	
Breast cancer	358 (10.8)	106 (5.5)		256 (11.1)	74 (5.4)		102 (10.1)	32 (5.8)	
Female reproductive cancer	104 (3.1)	73 (3.8)		76 (3.3)	54 (4.0)		28 (2.8)	19 (3.4)	
Urological cancer	69 (2.1)	63 (3.3)		45 (2.0)	45 (3.3)		24 (2.4)	18 (3.3)	
Nasopharyngeal cancer	94 (2.8)	32 (1.7)		69 (3.0)	23 (1.7)		25 (2.5)	9 (1.6)	
Other cancer	79 (2.4)	77 (4.0)		56 (2.4)	57 (4.2)		23 (2.3)	20 (3.6)	
Tumor stage (%)									
I	331 (10.0)	110 (5.8)	<0.001	241 (10.5)	79 (5.8)	<0.001	90 (8.9)	31 (5.6)	<0.001
II	715 (21.6)	228 (11.9)		508 (22.1)	150 (11.0)		207 (20.4)	78 (14.2)	
III	971 (29.3)	442 (23.1)		667 (29.0)	313 (23.0)		304 (30.0)	129 (23.4)	
IV	1294 (39.1)	1130 (59.2)		882 (38.4)	817 (60.1)		412 (40.7)	313 (56.8)	
Surgery, yes (%)	1838 (55.5)	758 (39.7)	<0.001	1281 (55.7)	546 (40.2)	<0.001	557 (55.0)	212 (38.5)	<0.001
Radiotherapy, yes (%)	365 (11.0)	216 (11.3)	0.787	258 (11.2)	146 (10.7)	0.692	107 (10.6)	70 (12.7)	0.233
Chemotherapy, yes (%)	2099 (63.4)	1213 (63.5)	0.959	1450 (63.1)	867 (63.8)	0.698	649 (64.1)	346 (62.8)	0.657
KPS (mean (SD))	87.24 (10.41)	82.20 (14.60)	<0.001	87.30 (10.48)	82.07 (14.89)	<0.001	87.12 (10.25)	82.52 (13.87)	<0.001
KPS (%)									
≥70	3195 (96.5)	1719 (90.0)	<0.001	2211 (96.2)	1219 (89.7)	<0.001	984 (97.1)	500 (90.7)	<0.001
<70	116 (3.5)	191 (10.0)		87 (3.8)	140 (10.3)		29 (2.9)	51 (9.3)	
PGSGA (mean (SD))	5.25 (4.20)	7.33 (5.33)	<0.001	5.21 (4.22)	7.40 (5.28)	<0.001	5.33 (4.16)	7.15 (5.47)	<0.001
Nutritional intervention, yes (%)	574 (17.3)	426 (22.3)	<0.001	409 (17.8)	313 (23.0)	<0.001	165 (16.3)	113 (20.5)	0.044
TSF, mm (mean (SD))	16.17 (8.66)	16.28 (9.18)	0.674	16.12 (8.60)	16.24 (9.14)	0.674	16.30 (8.78)	16.37 (9.28)	0.883
TC, mmol/L (mean (SD))	4.60 (1.04)	4.58 (1.24)	0.543	4.60 (1.03)	4.57 (1.23)	0.436	4.61 (1.06)	4.62 (1.27)	0.923
Blood glucose, mmol/L (mean (SD))	5.47 (1.21)	6.31 (2.35)	<0.001	5.48 (1.24)	6.30 (2.27)	<0.001	5.47 (1.14)	6.32 (2.54)	<0.001
CRP, mg/L (median (IQR))	3.02 (2.45)	25.00 (43.37)	<0.001	3.02 (2.41)	25.00 (42.34)	<0.001	3.02 (2.15)	25.00 (30.87)	<0.001
TyG (mean (SD))	3.82 (0.25)	3.99 (0.33)	<0.001	6.30 (2.27)	3.99 (0.33)	<0.001	3.81 (0.25)	3.99 (0.33)	<0.001
CTI (mean (SD))	4.15 (0.54)	5.31 (0.40)	<0.001	4.14 (0.55)	5.31 (0.40)	<0.001	4.18 (0.51)	5.31 (0.39)	<0.001

Low CTI, CTI ≤ 4.78; High CTI, CTI>4.78. SD, standard deviation; IQR, interquartile range; BMI, body mass index; KPS, karnofsky performance status; PGSGA, patient-generated subjective global assessment; TC, total cholesterol; CRP, C-reactive protein; CTI, C-reactive protein-triglyceride glucose index.

### Correlation Analysis and Distribution of CTI

We performed a correlation analysis between CTI and different prognostic parameters, and the results showed that CTI was significantly positively correlated with CRP (correlation coefficient = 0.627, *P* < 0.001) and TyG (correlation coefficient = 0.360, *P* < 0.001), whereas CTI and glucose (correlation coefficient = 0.269, *P* < 0.001), TNM stage (correlation coefficient = 0.214, *P* < 0.001), KPS (correlation coefficient = –0.192, *P* < 0.001) and PGSGA (correlation coefficient = 0.205, *P* < 0.001) showed a significantly weak correlation. Similarly, the results were relatively consistent in different age and sex groups (**Figures S3**, **S4**).

**Figure 1:**
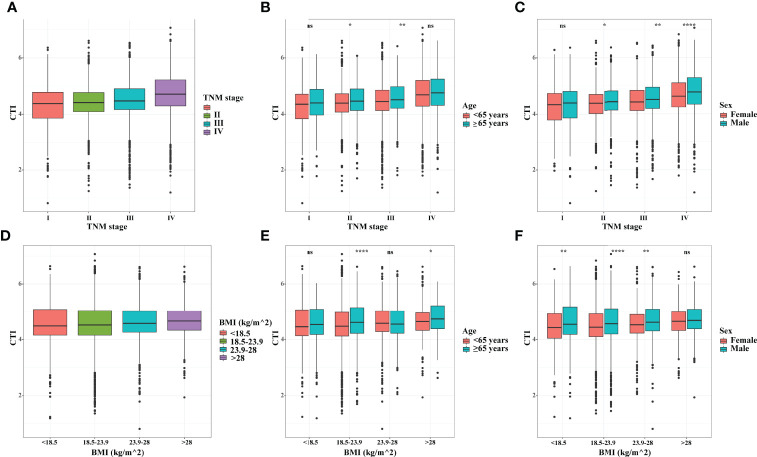
The distribution of CTI in different groups. **(A)** CTI in TNM stage groups; **(B)** CTI in TNM stage groups stratified by age; **(C)** CTI in TNM stage groups stratified by sex; **(D)** CTI in BMI groups; **(E)** CTI in BMI groups stratified by age; **(F)** CTI in BMI groups stratified by sex. CTI, C-reactive protein-triglyceride glucose index; BMI, body mass index. *, P<0.05; **, P<0.01; ****,P<0.0001; ns, not significant.

The distribution of CTI in different subgroups showed an upward trend in CTI with TNM stage progression and BMI increase. Interestingly, in different age and sex groups, the CTI levels of male patients or elderly patients (≥65years) were compared with those of female patients or younger patients (<65 years), respectively. (**Figure 1**). Furthermore, we also analyzed the distribution of CTI in different age groups (As a categorical variable and a continuous variable), and the results showed that the value of CTI increased with age (**Figure S5**).

The distribution curves of CTI in different tumor type subgroups showed good agreement among different tumor type subgroups (except breast cancer and nasopharyngeal cancer) (**Figure S6**).

### Prognostic Effect of CTI in Patients With Cancer

**Figure 2:**
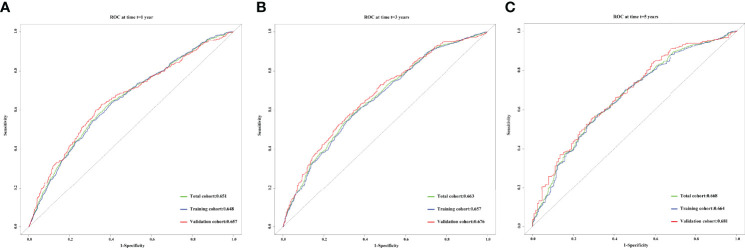
The 1-, 3-, and 5-year prognostic ROC curves of CTI in the different cohorts of patients with cancer. **(A)** 1- year prognostic ROC curve; **(B)** 3- year prognostic ROC curve; **(C)** 5- year prognostic ROC curve. Green line, total cohort; Blue line, training cohort; Red line, validation cohort. CTI, C-reactive protein-triglyceride glucose index; ROC, receiver operating characteristic.

**Figure 3:**
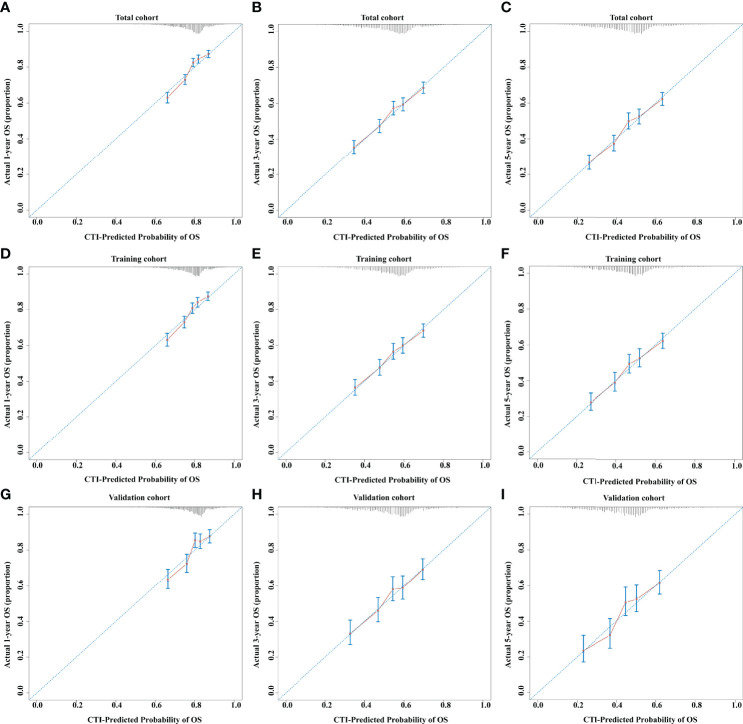
The 1-, 3-, and 5-year calibration curves of CTI in the different cohorts of patients with cancer. **(A–C)** 1-, 3-, and 5-year calibration curves of CTI in total cohort. **(D–F)** 1-, 3-, and 5-year calibration curves of CTI in training cohort. **(G–)** 1-, 3-, and 5-year calibration curves of CTI in validation cohort. CTI, C-reactive protein-triglyceride glucose index.

The concordance (C)-indices of CTI in patients with cancer were 0.636 (0.624–0.624), 0.617 (0.602–0.633), and 0.631 (0.608–0.655) in the total, training, and validation cohorts, respectively. We performed survival prediction on the prognosis of CTI in patients with cancer, and the results showed that CTI at 1-year (total cohort, 0.651; training cohort, 0.648; validation cohort, 0.657), 3-year (total cohort, 0.663; training cohort, 0.657; validation cohort, 0.676), and 5-year (total cohort, 0.668; training cohort, 0.664; validation cohort, 0.681) ROCs showed good survival predictors in patients with cancer (**Figure 2**). Meanwhile, in the total, training, and validation cohorts, the 1-, 3-, and 5-year calibration curve results showed that CTI had good survival prediction consistency for patients with cancer (**Figure 3**).

**Figure 4:**
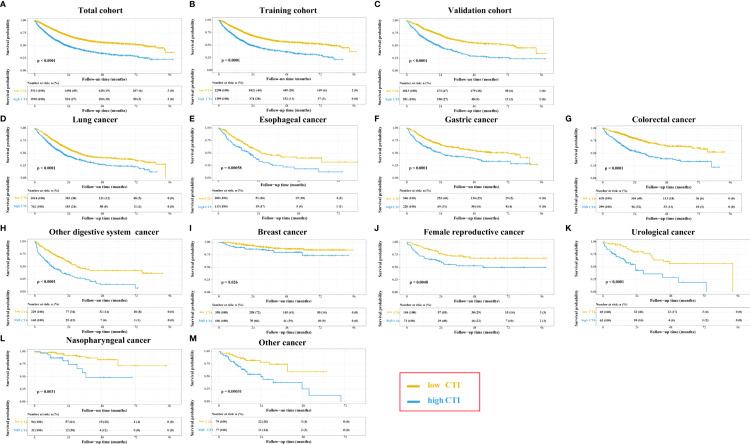
The Kaplan-Meier survival curves of CTI in the different cohorts of patients with cancer. **(A)** Total cohort; **(B)** Training cohort; **(C)** Validation cohort; **(D–M)** Different tumor types based on total cohort: **(D)**Lung cancer; **(E)** Esophageal cancer; **(F)** Gastric cancer; **(G)** Colorectal cancer; **(H)** Other digsestive cancer; **(I)** Breast cancer; **(J)** Female reproductive cancer; **(K)** Urological cancer; **(L)** Nasopharyngeal cancer; **(M)** Other cancer; CTI, C-reactive protein-triglyceride glucose index.

In the total cohort, the survival curve of CTI showed that patients with high CTI predicted a worse prognosis (*P* < 0.0001) (**Figure 4A**). The restricted cubic spline of CTI in the survival of patients with cancer showed tha the risk of death in patients with cancer increased with increasing CTI (Adjusted for model 3) (**Figure S7**). The multivariate survival showed that each SD increased in CTI was associated with a 22% increase in the risk of death (adjusted for model 3: *P* < 0.001, HR=1.22, 95% CI = 1.17-1.28). Compared with patients with low CTI, the prognosis of patients with high CTI was worse (adjusted for model 3: *P* < 0.001, HR = 1.46, 95% CI = 1.33–1.59). Similarly, we divided CTI into four groups, and compared with patients in the Q1 group, patients in the Q3 (adjusted for model 3: *P* < 0.001, HR = 1.36, 95% CI = 1.20–1.55), and Q4 (adjusted for model 3: *P* < 0.001, HR = 1.72, 95% CI = 1.51–1.95) groups had worse OS in patients with cancer (**Table 3**). We performed a sensitivity analysis of the total cohort, and the results remained consistent (**Table S1**).

**Table 3:** Univariate and multivariate analysis of CTI in total, training, and validation cohort.

Variables	OS (model 0) ^a^		OS (model 1) ^b^		OS (model 2) ^c^		OS (model 3) ^d^	
	Crude HR (95%CI)	Crude *P*	Adjusted HR (95%CI)	Adjusted *P*	Adjusted HR (95%CI)	Adjusted *P*	Adjusted HR (95%CI)	Adjusted *P*
**Total cohort**								
As continues (per SD)	1.44 (1.38-1.50)	<0.001	1.30 (1.24-1.35)	<0.001	1.22 (1.17-1.27)	<0.001	1.22 (1.17-1.28)	<0.001
By cutoff								
CTI ≤ 4.78	1		1		1		1	
CTI>4.78	2.04 (1.87-2.22)	<0.001	1.63 (1.50-1.78)	<0.001	1.45 (1.33-1.59)	<0.001	1.46 (1.33-1.59)	<0.001
As quartile								
Q1(<4.20)	1		1		1		1	
Q2(4.20-4.54)	1.22 (1.06-1.39)	0.004	1.16 (1.01-1.32)	0.032	1.12 (0.98-1.29)	0.087	1.13 (0.99-1.30)	0.069
Q3 (4.54-5.04)	1.64 (1.44-1.86)	<0.001	1.45 (1.27-1.64)	<0.001	1.35 (1.19-1.54)	<0.001	1.36 (1.20-1.55)	<0.001
Q4 (>5.04)	2.68 (2.38-3.02)	<0.001	2.03 (1.79-2.29)	<0.001	1.70 (1.50-1.93)	<0.001	1.72 (1.51-1.95)	<0.001
*P* for trend		<0.001		<0.001		<0.001		<0.001
**Training cohort**								
As continues (per SD)	1.43 (1.36-1.51)	<0.001	1.27 (1.21-1.34)	<0.001	1.20 (1.14-1.27)	<0.001	1.20 (1.14-1.27)	<0.001
By cutoff								
CTI ≤ 4.78	1		1		1		1	
CTI>4.78	1.98 (1.79-2.20)	<0.001	1.54 (1.39-1.71)	<0.001	1.36 (1.22-1.51)	<0.001	1.36 (1.22-1.52)	<0.001
As quartile								
Q1(<4.20)	1		1		1		1	
Q2(4.20-4.54)	1.23 (1.05-1.44)	0.011	1.13 (0.97-1.33)	0.126	1.11 (0.94-1.30)	0.214	1.11 (0.94-1.30)	0.212
Q3 (4.54-5.04)	1.63 (1.40-1.90)	<0.001	1.37 (1.17-1.59)	<0.001	1.25 (1.07-1.47)	0.004	1.26 (1.07-1.47)	0.004
Q4 (>5.04)	2.65 (2.30-3.06)	<0.001	1.92 (1.66,2.23)	<0.001	1.60 (1.38-1.87)	<0.001	1.61 (1.38-1.87)	<0.001
*P* for trend		<0.001		<0.001		<0.001		<0.001
**Validation cohort**								
As continues (per SD)	1.43 (1.33-1.55)	<0.001	1.36 (1.26-1.47)	<0.001	1.29 (1.19-1.40)	<0.001	1.30 (1.20-1.40)	<0.001
By cutoff								
CTI ≤ 4.78	1		1		1		1	
CTI>4.78	2.18 (1.86-2.54)	<0.001	1.90 (1.62-2.22)	<0.001	1.72 (1.46-2.03)	<0.001	1.73 (1.47-2.04)	<0.001
As quartile								
Q1(<4.20)	1		1		1		1	
Q2(4.20-4.54)	1.18 (0.93-1.50)	0.005	1.22 (0.96-1.55)	0.111	1.19 (0.93-1.52)	0.171	1.22 (0.96-1.57)	0.110
Q3 (4.54-5.04)	1.65 (1.31-2.08)	<0.001	1.65 (1.31-2.09)	<0.001	1.66 (1.31-2.11)	<0.001	1.71 (1.34-2.17)	<0.001
Q4 (>5.04)	2.76 (2.22-3.43)	<0.001	2.30 (1.84-2.88)	<0.001	2.00 (1.58-2.52)	<0.001	2.05 (1.62-2.58)	<0.001
*P* for trend		<0.001		<0.001		<0.001		<0.001

HR, hazards ratio; CI, confidence interval; BMI, body mass index; KPS, karnofsky performance status; CTI, C-reactive protein-triglyceride glucose index; TSF, skin-fold thickness.

a Model 0: Unadjusted.

b Model 1: Adjusted for age, sex, BMI and TNM stage.

c Model 2: Adjusted for age, sex, TNM stage, BMI, tumor types, KPS, surgery, chemotherapy, radiotherapy, smoking, alcohol, nutritional intervention, hypertension, coronary heart disease, and diabetes.

d Model 3: Adjusted for age, sex, TNM stage, BMI, tumor types, KPS, surgery, chemotherapy, radiotherapy, smoking, alcohol, nutritional intervention, hypertension, coronary heart disease, diabetes, and TSF.

In the training cohort, the survival curve of CTI showed that patients with high CTI predicted a worse prognosis (*P* < 0.0001) (**Figure 4B**). The multivariate survival showed that each SD increased in CTI was associated with a 20% increase in the risk of death (adjusted for model 3: *P* < 0.001, HR=1.20, 95% CI = 1.14–1.27). Compared with patients with low CTI, the prognosis of patients with high CTI was worse (adjusted for model 3: *P* < 0.001, HR = 1.36, 95% CI = 1.22–1.52). Similarly, we divided CTI into four groups, and compared with patients in the Q1 group, patients in the Q3 (adjusted for model 3: *P* < 0.001, HR = 1.26, 95% CI = 1.07–1.47) and Q4 (adjusted for model 3: *P* < 0.001, HR = 1.61, 95% CI = 1.38–1.87) groups had worse OS in patients with cancer (**Table 3**).

In the validation cohort, the survival curve of CTI showed that patients with high CTI predicted a worse prognosis (*P* < 0.0001) (**Figure 4C**). The multivariate survival showed that each SD increased in CTI was associated with an 30% increase in the risk of death (adjusted for model 3: *P* < 0.001, HR=1.30, 95% CI = 1.20–1.40). Compared with patients with low CTI, the prognosis of patients with high CTI was worse (adjusted for model 3: *P* < 0.001, HR = 1.73, 95% CI = 1.47–2.04). Similarly, we divided CTI into four groups, and compared with patients in the Q1 group, patients in the Q3 (adjusted for model 3: *P* < 0.001, HR = 1.71, 95% CI = 1.34–2.17), and Q4 (adjusted for model 3: *P* < 0.001, HR = 2.05, 95% CI = 1.62–2.58) groups had worse OS in patients with cancer (**Table 3**).

### Survival Analysis of Different Tumor Types

In the total cohort, patients with cancer in that of lung, esophageal, gastric, colorectal, other digestive system, breast, female reproductive, urological, nasopharyngeal, and other cancers showed that patients with high CTI had significantly worse prognosis than those with low CTI (all *P* < 0.05) (**Figure 4D–M**). In the training cohort, patients with high CTI in all cancer types, except for breast cancer, showed significantly poorer survival differences (all *P* < 0.05) (**Figure S8**). In the validation cohort, patients with high CTI in all cancer types, except for breast cancer and other cancer, showed significantly poorer survival differences (all *P* < 0.05) (**Figure S9**).

### Subgroup Analysis

**Figure 5:**
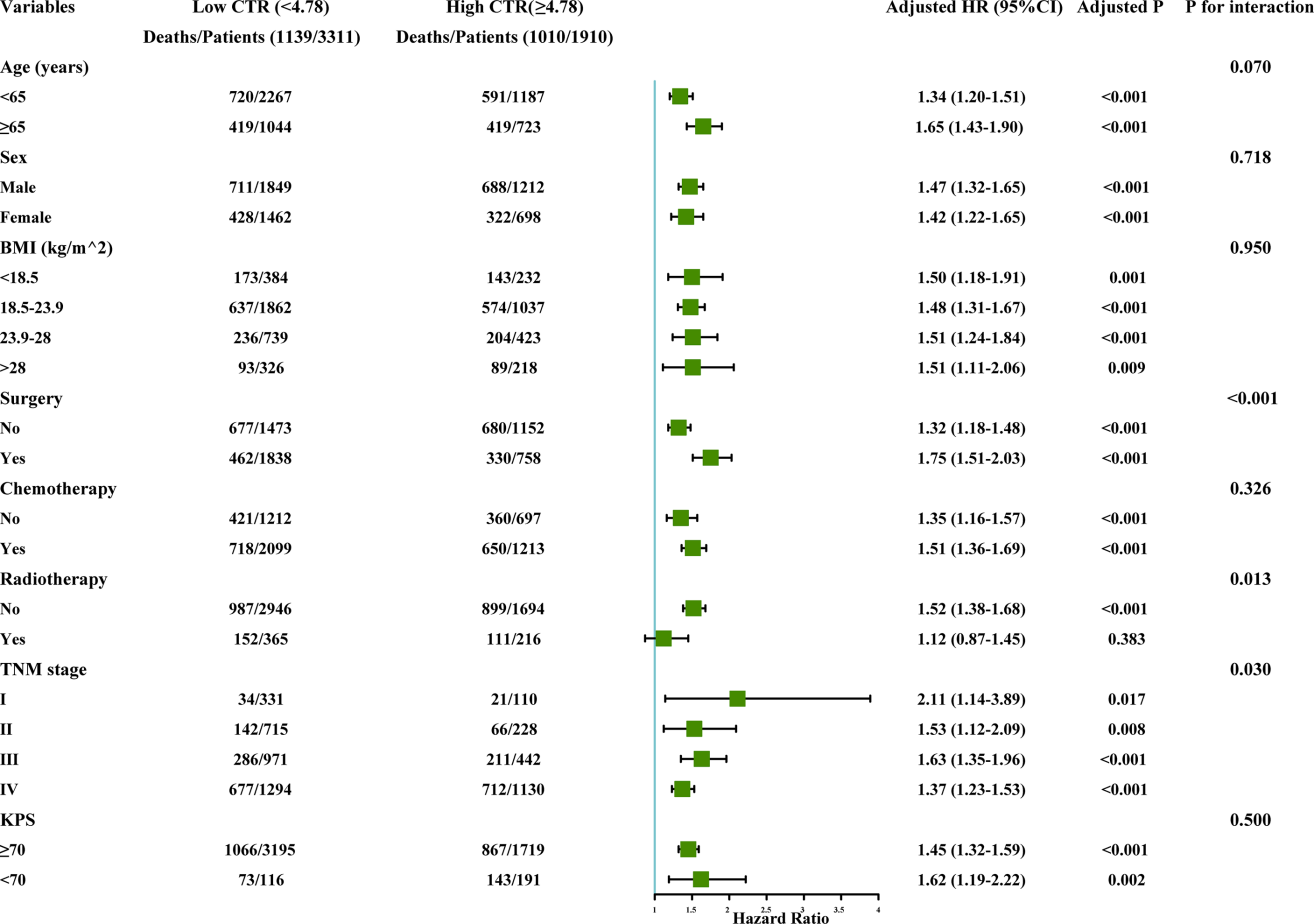
The subgroup analysis of the CTI in the total cohort of patients with cancer. Adjusted for age, sex, TNM stage, BMI, tumor types, KPS, surgery, chemotherapy, radiotherapy, smoking, alcohol, nutritional intervention, hypertension, coronary heart disease, diabetes, and TSF. CTI, C-reactive protein-triglyceride glucose index; BMI, body mass index; KPS, karnofsky performance status; TSF, triceps skin fold.

Subgroup analysis showed that CT had a significant interaction with TNM stage (*P* interaction = 0.030) radiotherapy (*P* interaction = 0.013), and surgery (*P* interaction < 0.001). However, no significant interaction was observed for CTI among age, sex, BMI, chemotherapy, radiotherapy, and KPS (**Figure 5**).

## Discussion

This study was designed to develop and validate an inflammation- and IR-related survival predictor in patients with cancer. We found that patients with advanced stage (III–IV) and low BMI (< 18.5 kg/m^2^) had higher CTI values, indicating higher levels of inflammation and IR. The risk of cancer progression varies and depends on a variety of factors, such as cancer type and stage, presence of systemic inflammation, low food intake, and lack of response to anticancer therapy (28). Later staging often predicts poor survival, with cachexia in up to 80% of patients with advanced cancer (29). In refractory cachexia, cachexia may be clinically refractory due to the presence of significantly advanced (advanced stage) or rapidly progressive cancer that is not responsive to anticancer therapy (28). A review found that in most clinical studies, cancer cachexia could be defined as BMI <18.5 kg/m^2^ (13). Thus, patients with low BMI have a high risk of cachexia. Therefore, we hypothesized that high CTI values in patients with advanced stage and patients with low BMI might be associated with a high risk of cancer cachexia. Cachexia-related inflammation is the result of a variety of changes, one of which is the pro-inflammatory factors secreted by the tumor itself. Systemic inflammation is a hallmark of patients with cancer. The inflammatory response is the main driving force behind the metabolic alterations observed in cancer (30). Persistent inflammatory mediators in patients with cancer can promote cancer cachexia, which in turn promotes IR (21, 22). In addition, we also found that with the increase of age, the proportion of high CTI in older patients with cancer also increased. Older patients with cancer experienced a higher level of inflammation and IR.

The survival analysis of CTI in patients with cancer showed that CTI had a good and consistent prognostic value in the total, training, and validation cohorts. Different adjustment models were used in this study, and we found that the risk of death of patients did not significantly change after the adjustment model added TSF. In some patients, inflammation leads to anorexia and fat loss, along with skeletal muscle loss. In other cases, appetite and food intake remain unchanged despite the activation of systemic inflammation, resulting in sarcopenia associated with normal or elevated BMI (31). Interestingly, our BMI distribution diagram showed that CTI was relatively high in patients with low BMI. Recent epidemiological evidence suggests that the deleterious effects of low BMI on outcomes are primarily due to the deleterious effects of muscle loss and dysfunction (32). The systemic inflammatory response involves stimulation of multiple mediators, primarily cortisol and pro-inflammatory cytokines, that can directly activate the ubiquitin-proteasome pathway and autophagy in skeletal muscle and inhibit myofibrillar protein synthesis (31). Skeletal muscle is the primary site of dietary glucose processing; therefore, muscle sensitivity to insulin action is critical for the development of systemic IR and hyperglycemia (33). Deficiencies in muscle mass may also lead to IR. A large epidemiological study showed that skeletal muscle mass and insulin sensitivity were significantly inversely associated with bioimpedance and homeostasis model assessments, respectively (34). Therefore, we hypothesized that it might not be the body fat but the muscle mass that affected the survival of patients with cancer.

In our sensitivity analysis, we performed a survival analysis of CTI in the non-diabetic and diabetic populations and found that CTI predicted a higher HR and a worse prognosis in the diabetic population than in the non-diabetic population. We compared CTI values in diabetic and non-diabetic subjects and found that diabetic subjects had higher CTI than non-diabetic subjects (**Figure S10**). IR is closely related to T2D. Systemic inflammation can play an important role in the occurrence of IR (23). Inflammatory cytokines, such as IL-1β and interferon-γ, can regulate insulin signaling (23, 35). Interestingly, hyperinsulinemia induces the increase in inflammatory cytokines, IL-6 and TNF-α, thereby promoting IR (4). Although blood glucose is not an indicator for judging the IR status of patients, timely monitoring and regulation of inflammation and IR status of the body are required when patients with cancer are accompanied by diabetes. Subgroup analysis showed a significant interaction between CTI and TNM stage, radiotherapy, and surgical treatment. In addition to paying attention to the CTI of advanced patients with cancer, CTI of early patients with cancer should also be considered. The results showed that patients with high CTI in the early stage had the highest risk of death among patients in other groups. In addition, high CTI in patients receiving radiotherapy and surgical treatment increased the risk of death in patients, suggesting that careful attention should be paid to the inflammation and IR status of patients with cancer when evaluating patients for radiotherapy and surgical treatment in clinical practice. These results still need to be further confirmed.

To the best of our knowledge, this is the first study to develop and validate an inflammation- and IR-related marker in patients with cancer based on CRP and TyG. However, some limitations need to be considered. First, this study only selected TyG as a simple surrogate index for IR, which may have a certain effect on the results. It is worth mentioning that there are many other IR indicators that need to be further collected. Second, this study only used an internal cohort for validation, and an external cohort is required to further validate this result. Third, CTI was composed of CRP and TyG. We found that CTI was highly correlated with CRP, but after the consistency test, the two indices were moderately consistent, and CTI was different from CRP and TyG. Of course, more clinical samples might be needed for validation. Fourth, although this is a large study, the study population is heterogeneous and studies focusing on more homogeneous tumor types are needed to compare the prognostic significance of CTI and prognostic parameters associated with individual tumor types. Finally, this was a cross-sectional study, and it may be necessary to dynamically observe the inflammatory and IR status in patients with cancer, as surgery and chemoradiotherapy may affect the status of patients with cancer.

## Conclusion

In conclusion, our study developed and validated the prognostic value of inflammation- and IR-related CTI in patients with cancer. Moreover, patients with cancer with high CTI predicted a worse prognosis. CTI is a simple and well-defined OS indicator for patients with cancer, which will allow clinicians to assess patients’ inflammatory and IR status and tailor treatment.

## Data Availability Statement

The raw data supporting the conclusions of this article will be made available by the authors, without undue reservation.

## Ethics Statement

This study followed the Declaration of Helsinki. All participants signed an informed consent form, and this study involving human participants was reviewed and approved by the institutional review board of each hospital(registration number: ChiCTR1800020329). The patients/participants provided their written informed consent to participate in this study.

## Author Contributions

G-TR wrote the manuscript. G-TR, H-LX, H-YZ and analyzed and interpreted the patient data, G-TR, H-LX, H-YZ, and H-PS made substantial contributions to the conception, design, and intellectual content of the studies. All authors read and approved the final manuscript.

## Funding

This work was supported by the National Key Research and Development Program [grant number 2017YFC1309200] and the Beijing Municipal Science and Technology Commission [grant number SCW2018-06].

## Conflict of Interest

The authors declare that the research was conducted in the absence of any commercial or financial relationships that could be construed as a potential conflict of interest.

## Publisher’s Note

All claims expressed in this article are solely those of the authors and do not necessarily represent those of their affiliated organizations, or those of the publisher, the editors and the reviewers. Any product that may be evaluated in this article, or claim that may be made by its manufacturer, is not guaranteed or endorsed by the publisher.

## Supplementary Material

Supplementary Figure 1 Flowchart of patient selection for this study.

Supplementary Figure 2 Optimal cut-off value of CTI. CTI, C-reactive protein-triglyceride glucose index.

Supplementary Figure 3 The Pearson correlation analysis stratified by age. CTI, C-reactive protein-triglyceride glucose index; BMI, body mass index; CRP, C-reactive protein; TyG, triglyceride-glucose index; Tch, triglyceride; KPS, karnofsky performance status; PGSGA, patient-generated subjective global assessment.

Supplementary Figure 4 The Pearson correlation analysis stratified by sex. CTI, C-reactive protein-triglyceride glucose index; BMI, body mass index; CRP, C-reactive protein; TyG, triglyceride-glucose index; Tch, triglyceride; KPS, karnofsky performance status; PGSGA, patient-generated subjective global assessment.

Supplementary Figure 5 The distribution of CTI stratified by age in different groups. CTI, C-reactive protein-triglyceride glucose index.

Supplementary Figure 6 The distribution of CTI in different tumor type groups. CTI, C-reactive protein-triglyceride glucose index.

Supplementary Figure 7 The restricted cubic spline curves of CTI in patients with cancer. CTI, C-reactive protein-triglyceride glucose index.

Supplementary Figure 8 The Kaplan-Meier survival curves of CTI in the training cohort of patients with cancer. **(A)** Lung cancer; **(B)** Esophageal cancer; **(C)** Gastric cancer; **(D)** Colorectal cancer; **(E)** Other digsestive cancer; **(F)** Breast cancer; **(G)** Female reproductive cancer; **(H)** Urological cancer; **(I)** Nasopharyngeal cancer; **(J)** Other cancer; CTI, C-reactive protein-triglyceride glucose index.

Supplementary Figure 9 The Kaplan-Meier survival curves of CTI in the validation cohort of patients with cancer. **(A)** Lung cancer; **(B)** Esophageal cancer; **(C)** Gastric cancer; **(D)** Colorectal cancer; **(E)** Other digsestive cancer; **(F)** Breast cancer; **(G)** Female reproductive cancer; **(H)** Urological cancer; **(I)** Nasopharyngeal cancer; **(J)** Other cancer; CTI, C-reactive protein-triglyceride glucose index.

Supplementary Figure 10 The distribution of CTI in the diabetes and non-diabetes groups. CTI, C-reactive protein-triglyceride glucose index.
