# Supplementary figures and images for: A Novel Inflammation and Insulin Resistance Related Indicator to Predict the Survival of Patients With Cancer
Source: Front Endocrinol (Lausanne). 2022 Jun 20;13:905266. doi: 10.3389/fendo.2022.905266 (PMC9252441; doi:10.3389/fendo.2022.905266)

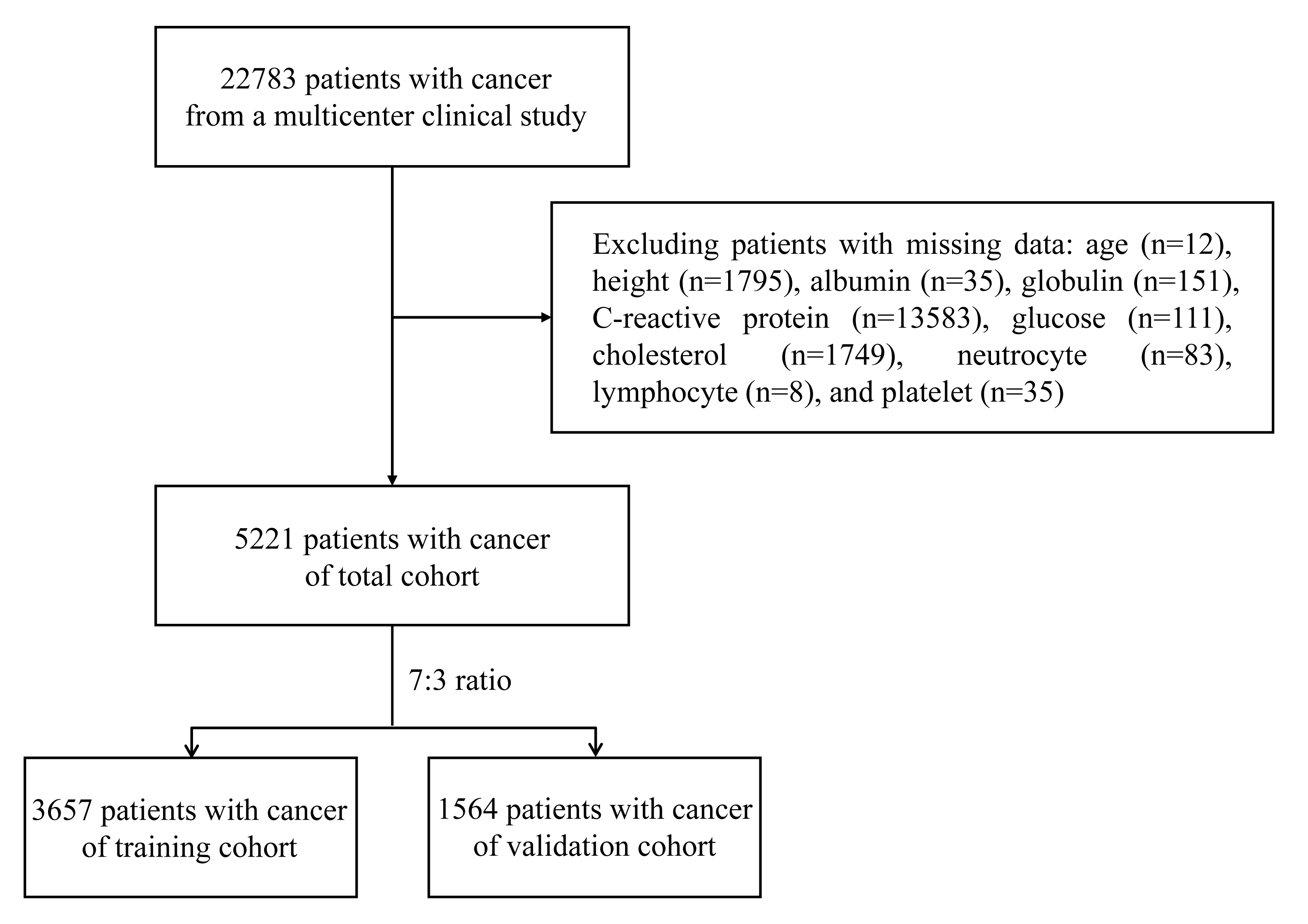

Supplement: Supplementary Figure 1 — Flowchart of patient selection for this study. [file Image_1.tif]

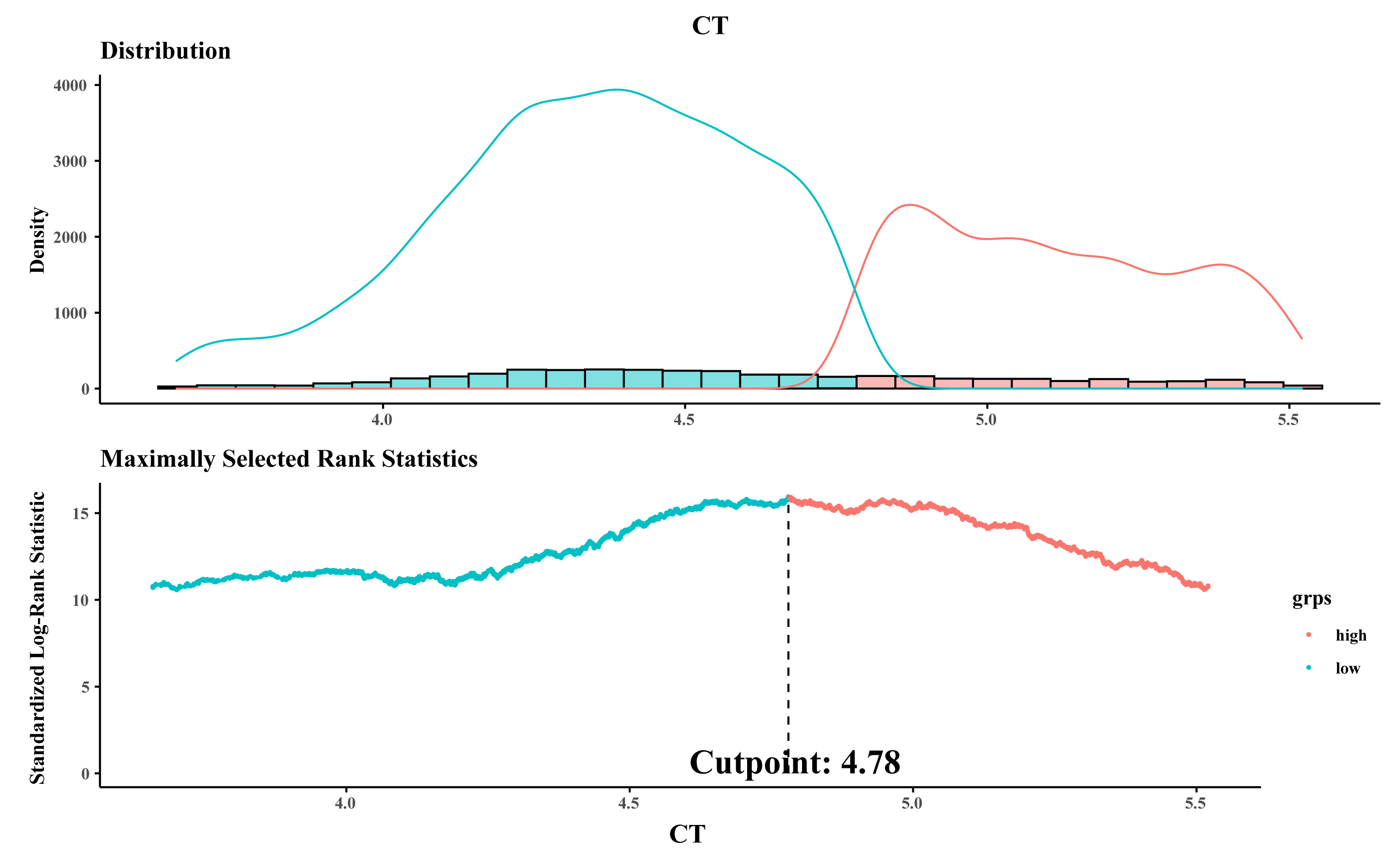

Supplement: Supplementary Figure 2 — Optimal cut-off value of CTI. CTI, C-reactive protein-triglyceride glucose index. [file Image_2.tif]

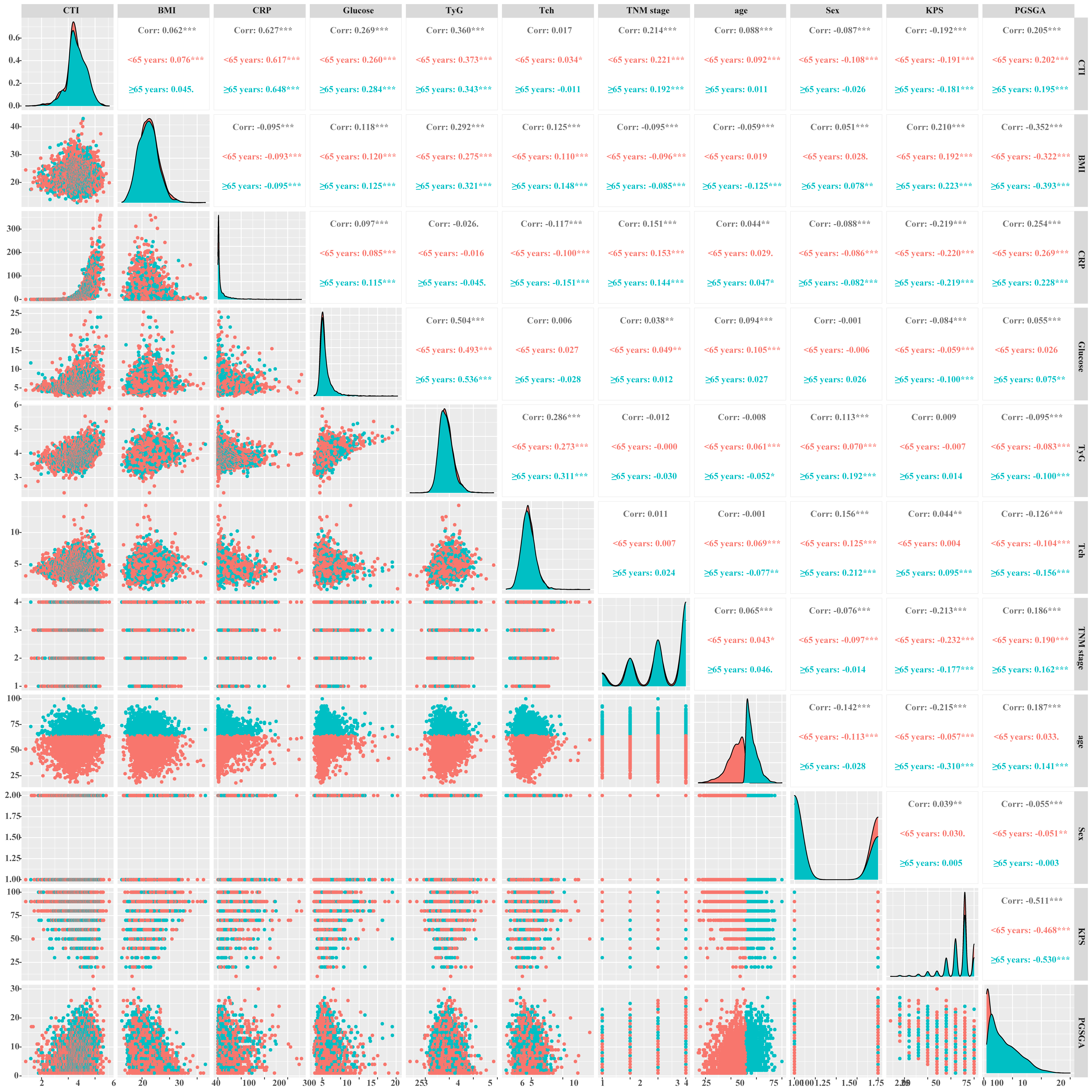

Supplement: Supplementary Figure 3 — The Pearson correlation analysis stratified by age. CTI, C-reactive protein-triglyceride glucose index; BMI, body mass index; CRP, C-reactive protein; TyG, triglyceride-glucose index; Tch, triglyceride; KPS, karnofsky performance status; PGSGA, patient-generated subjective global assessment. [file Image_3.tif]

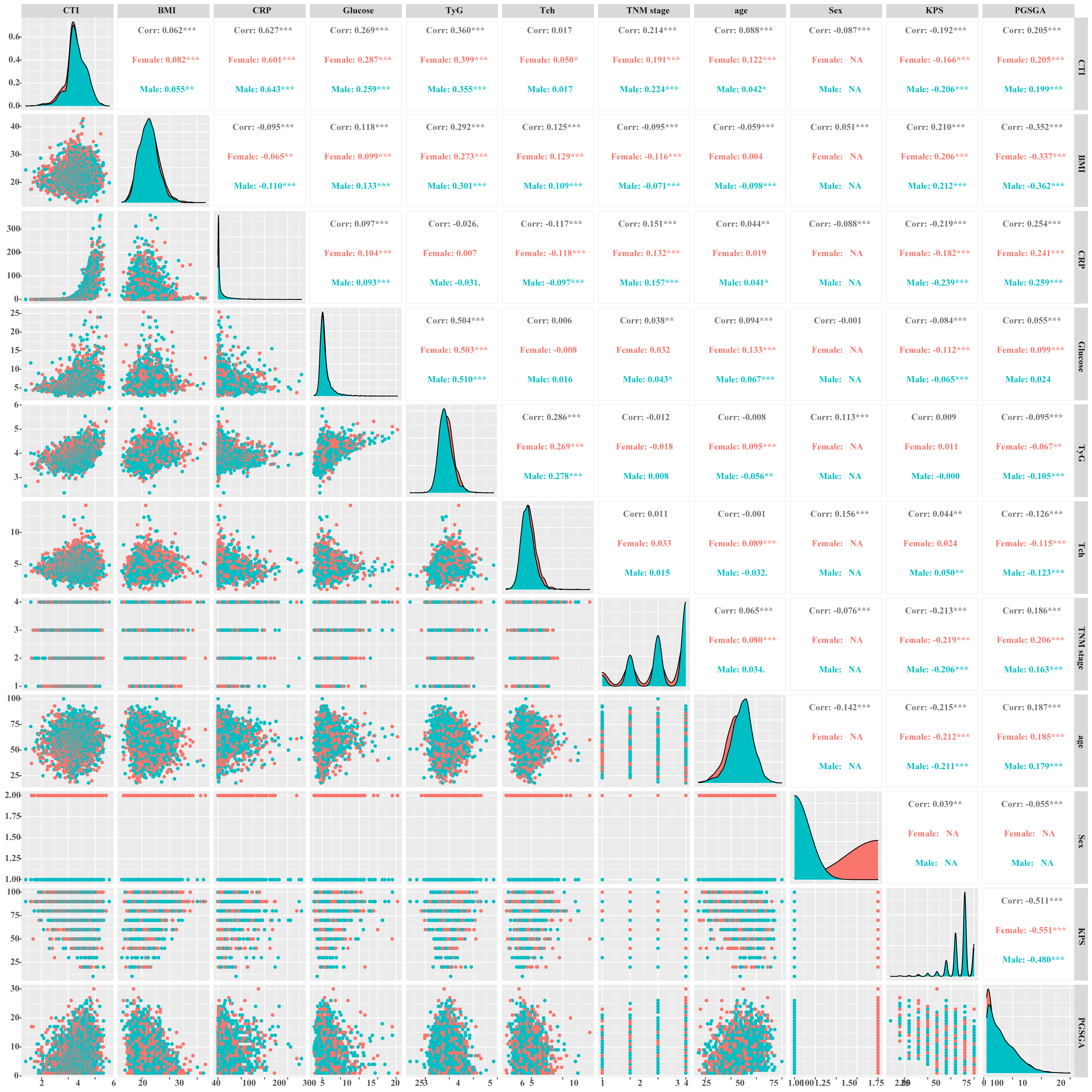

Supplement: Supplementary Figure 4 — The Pearson correlation analysis stratified by sex. CTI, C-reactive protein-triglyceride glucose index; BMI, body mass index; CRP, C-reactive protein; TyG, triglyceride-glucose index; Tch, triglyceride; KPS, karnofsky performance status; PGSGA, patient-generated subjective global assessment. [file Image_4.tif]

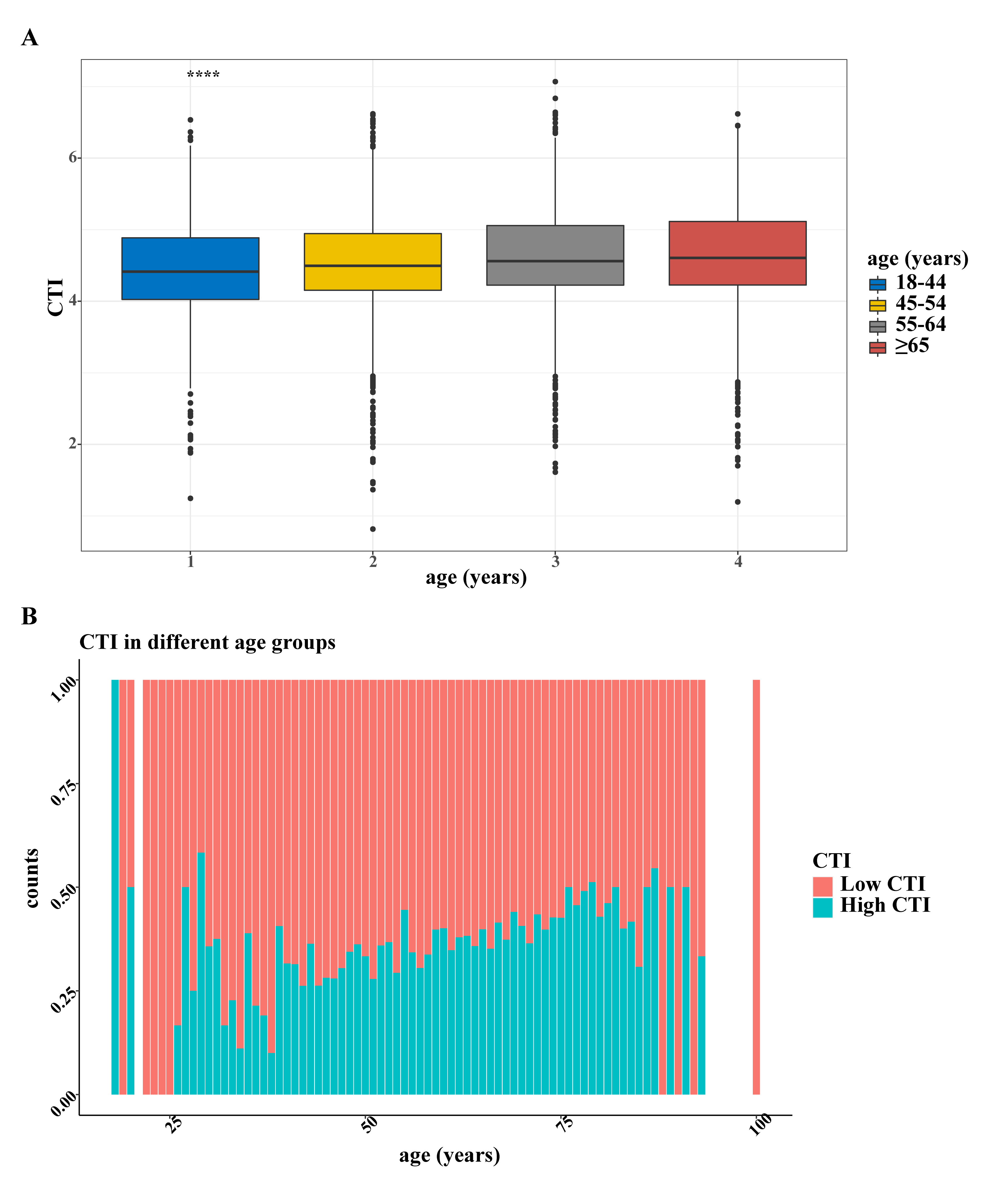

Supplement: Supplementary Figure 5 — The distribution of CTI stratified by age in different groups. CTI, C-reactive protein-triglyceride glucose index. [file Image_5.tif]

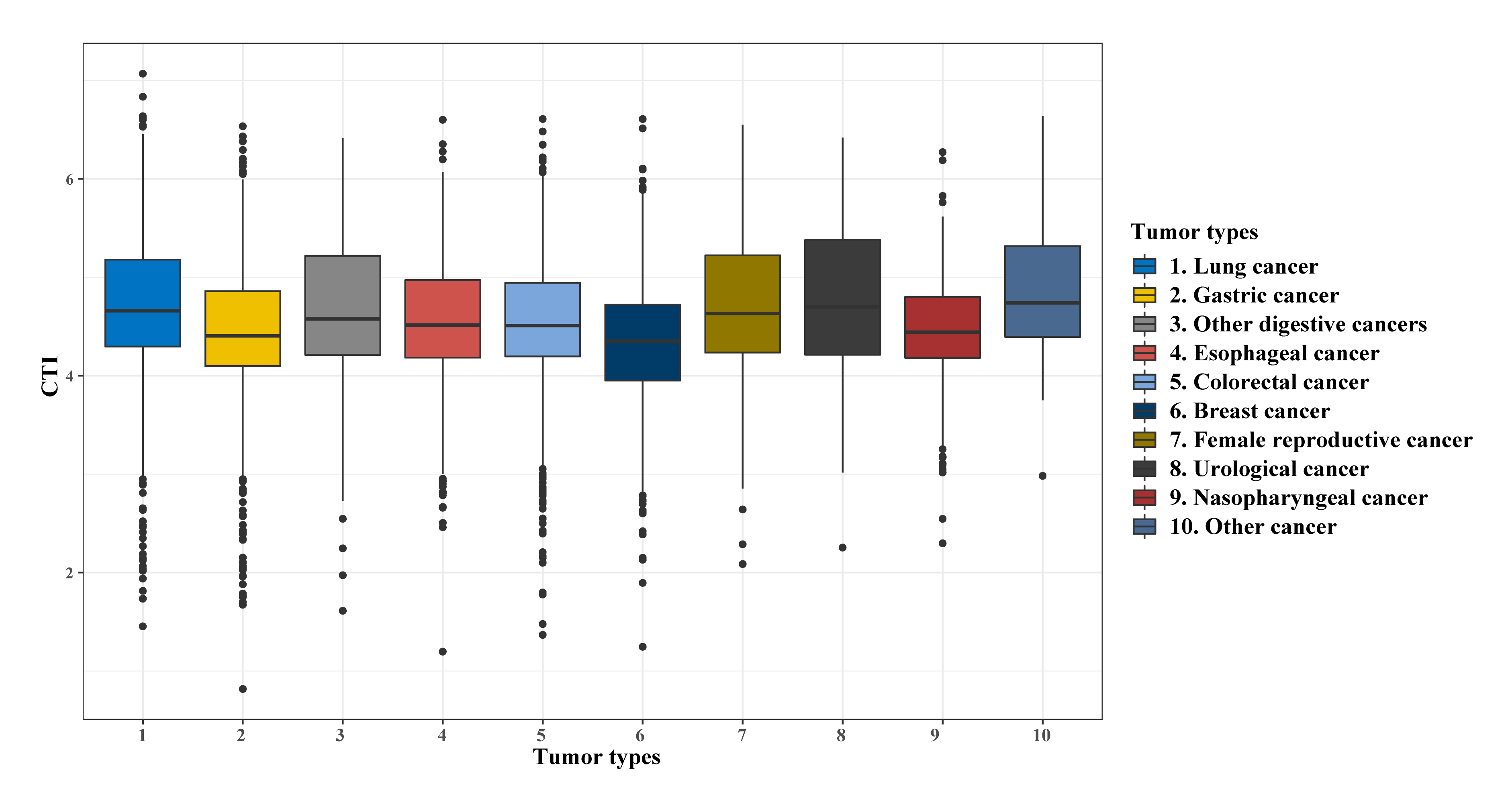

Supplement: Supplementary Figure 6 — The distribution of CTI in different tumor type groups. CTI, C-reactive protein-triglyceride glucose index. [file Image_6.tif]

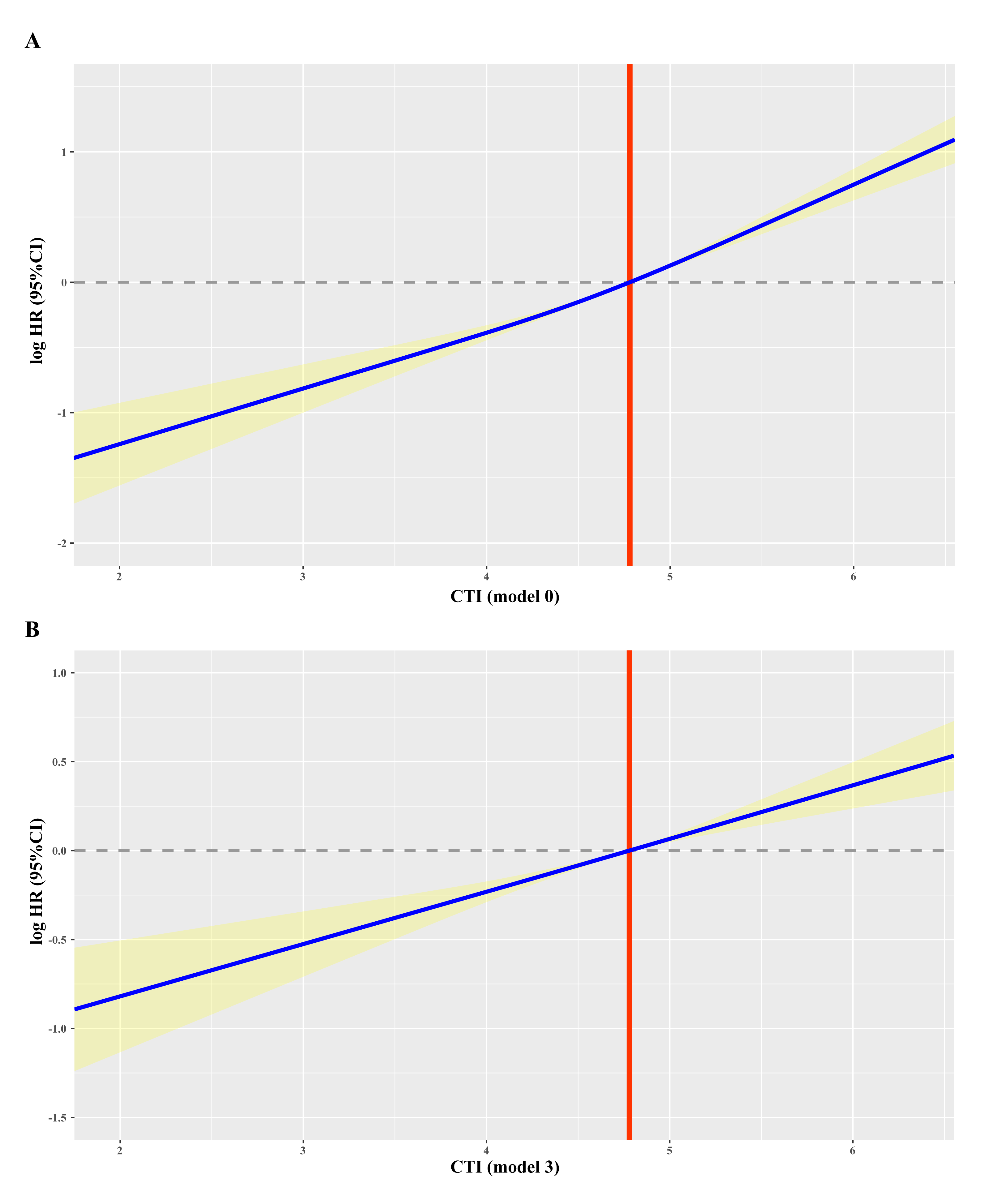

Supplement: Supplementary Figure 7 — The restricted cubic spline curves of CTI in patients with cancer. CTI, C-reactive protein-triglyceride glucose index. [file Image_7.tif]

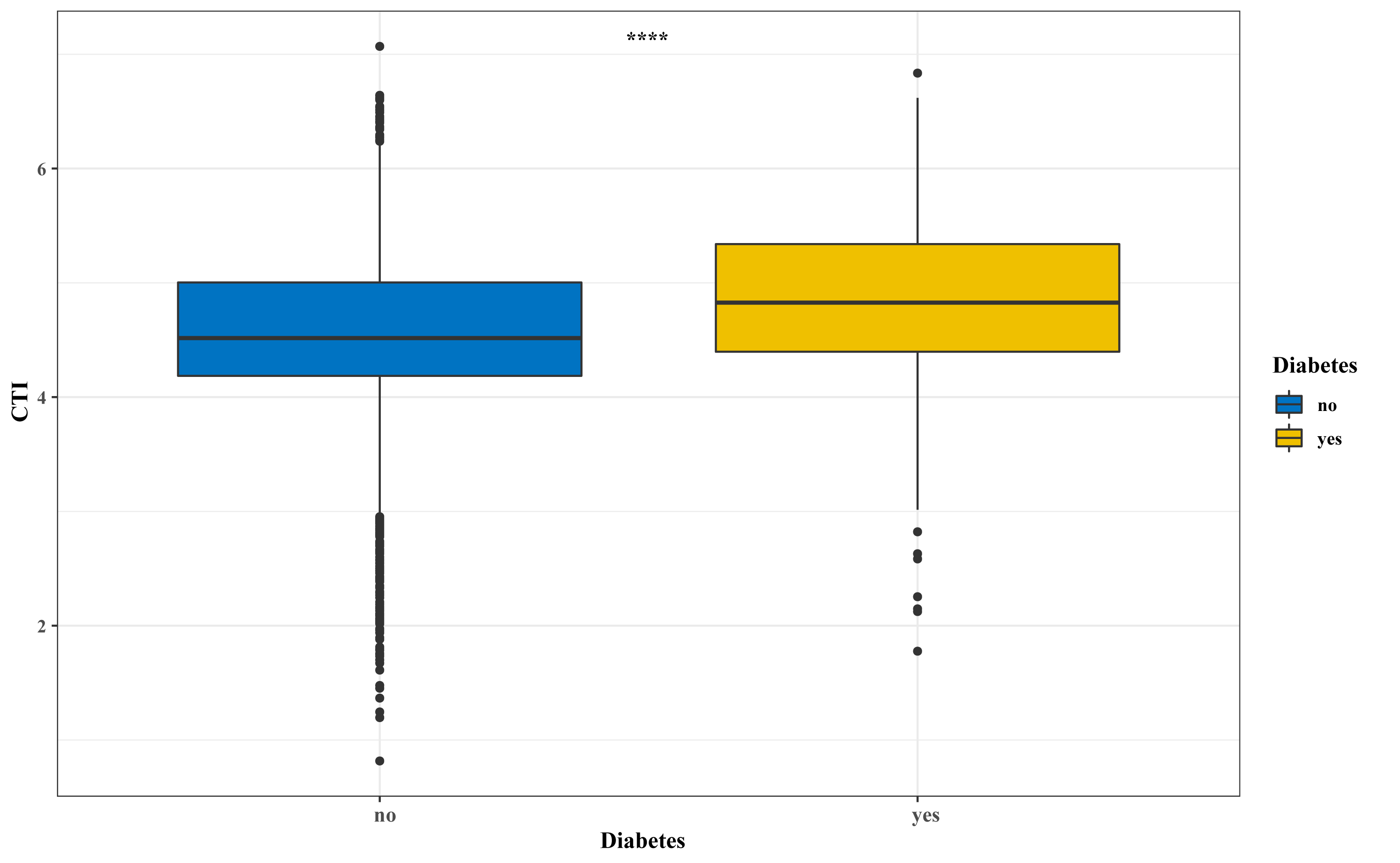

Supplement: Supplementary Figure 10 — The distribution of CTI in the diabetes and non-diabetes groups. CTI, C-reactive protein-triglyceride glucose index. [file Image_10.tif]
