# Supplementary material for: A Novel Inflammation and Insulin Resistance Related Indicator to Predict the Survival of Patients With Cancer
Source: Front Endocrinol (Lausanne). 2022 Jun 20;13:905266. doi: 10.3389/fendo.2022.905266 (PMC9252441; doi:10.3389/fendo.2022.905266)
Supplement: Supplementary file 11 [file Table_1.docx]

**Table S1 Sensitivity analysis.**

| Variables | OS ^a^ | |  | OS ^b^ | |  | OS ^c^ | |  | OS ^d^ | |
| --- | --- | --- | --- | --- | --- | --- | --- | --- | --- | --- | --- |
|  | Crude HR (95%CI) | Crude *P* |  | Adjusted HR (95%CI) | Adjusted *P* |  | Adjusted HR (95%CI) | Adjusted *P* |  | Adjusted HR (95%CI) | Adjusted *P* |
| **Patients who died within six months were excluded*** | | | | | | | | | | | |
| As continues (per SD) | 1.32 (1.26-1.39) | <0.001 |  | 1.19 (1.14-1.25) | <0.001 |  | 1.13 (1.08-1.19) | <0.001 |  | 1.13 (1.08-1.19) | <0.001 |
| By cutoff |  |  |  |  |  |  |  |  |  |  |  |
| CTI≤4.78 | 1 |  |  | 1 |  |  | 1 |  |  | 1 |  |
| CTI>4.78 | 1.77 (1.60-1.95) | <0.001 |  | 1.42 (1.29-1.58) | <0.001 |  | 1.28 (1.15-1.42) | <0.001 |  | 1.28 (1.15-1.42) | <0.001 |
| As quartile |  |  |  |  |  |  |  |  |  |  |  |
| Q1(<4.20) | 1 |  |  | 1 |  |  | 1 |  |  | 1 |  |
| Q2(4.20-4.54) | 1.12 (0.97-1.3) | 0.122 |  | 1.06 (0.91-1.23) | 0.001 |  | 1.03 (0.89-1.20) | 0.696 |  | 1.04 (0.89-1.20) | 0.641 |
| Q3 (4.54-5.04) | 1.54 (1.34-1.77) | <0.001 |  | 1.35 (1.17-1.56) | <0.001 |  | 1.27 (1.10-1.46) | 0.001 |  | 1.27 (1.10-1.47) | 0.001 |
| Q4 (>5.04) | 2.16 (1.88-2.48) | <0.001 |  | 1.63 (1.41-1.87) | <0.001 |  | 1.38 (1.19-1.59) | <0.001 |  | 1.38 (1.20-1.60) | <0.001 |
| *P* for trend |  | <0.001 |  |  | <0.001 |  |  | <0.001 |  |  | <0.001 |
| **Patients with diabetes were excluded^#^** | | | | | | | | | | | |
| As continues (per SD) | 1.43 (1.37-1.50) | <0.001 |  | 1.29 (1.23-1.35) | <0.001 |  | 1.15 (1.10-1.20) | <0.001 |  | 1.14 (1.10-1.20) | <0.001 |
| By cutoff |  |  |  |  |  |  |  |  |  |  |  |
| CTI≤4.78 | 1 |  |  | 1 |  |  | 1 |  |  | 1 |  |
| CTI>4.78 | 2.01 (1.83-2.2) | <0.001 |  | 1.60 (1.46-1.76) | <0.001 |  | 1.43 (1.30-1.57) | <0.001 |  | 1.43 (1.30-1.57) | <0.001 |
| As quartile |  |  |  |  |  |  |  |  |  |  |  |
| Q1(<4.20) | 1 |  |  | 1 |  |  | 1 |  |  | 1 |  |
| Q2(4.20-4.54) | 1.24 (1.08-1.42) | 0.002 |  | 1.18 (1.02-1.35) | 0.022 |  | 1.16 (1.01-1.34) | 0.033 |  | 1.17 (1.02-1.35) | 0.025 |
| Q3 (4.54-5.04) | 1.58 (1.38-1.81) | <0.001 |  | 1.41 (1.23-1.62) | <0.001 |  | 1.34 (1.17-1.53) | <0.001 |  | 1.35 (1.17-1.54) | <0.001 |
| Q4 (>5.04) | 2.65 (2.33-3.01) | <0.001 |  | 1.98 (1.74-2.25) | <0.001 |  | 1.68 (1.47-1.92) | <0.001 |  | 1.69 (1.48-1.93) | <0.001 |
| *P* for trend |  | <0.001 |  |  | <0.001 |  |  | <0.001 |  |  | <0.001 |
| **Patients with non-diabetes were excluded^&^** | | | | | | | | | | | |
| As continues (per SD) | 1.46 (1.30-1.66) | <0.001 |  | 1.33 (1.17-1.51) | <0.001 |  | 1.29 (1.13-1.49) | <0.001 |  | 1.32 (1.15-1.52) | <0.001 |
| By cutoff |  |  |  |  |  |  |  |  |  |  |  |
| CTI≤4.78 | 1 |  |  | 1 |  |  | 1 |  |  | 1 |  |
| CTI>4.78 | 2.06 (1.60-2.67) | <0.001 |  | 1.79 (1.38-2.32) | <0.001 |  | 1.75 (1.34-2.28) | <0.001 |  | 1.78 (1.36-2.33) | <0.001 |
| As quartile |  |  |  |  |  |  |  |  |  |  |  |
| Q1(<4.20) | 1 |  |  | 1 |  |  | 1 |  |  | 1 |  |
| Q2(4.20-4.54) | 0.93 (0.56-1.56) | 0.793 |  | 0.83 (0.50-1.40) | 0.490 |  | 0.76 (0.45-1.30) | 0.318 |  | 0.77 (0.45-1.31) | 0.332 |
| Q3 (4.54-5.04) | 1.99 (1.30-3.05) | 0.002 |  | 1.67 (1.08-2.58) | 0.002 |  | 1.57 (1.00-2.46) | 0.050 |  | 1.58 (1.01-2.48) | 0.046 |
| Q4 (>5.04) | 2.69 (1.78-4.05) | <0.001 |  | 2.11 (1.39-3.20) | <0.001 |  | 1.89 (1.22-2.92) | 0.004 |  | 1.99 (1.29-3.08) | 0.002 |
| *P* for trend |  | <0.001 |  |  | <0.001 |  |  | <0.001 |  |  | <0.001 |

Notes: * The sensitivity analysis was to exclude patients who died within 6 months. # The sensitivity analysis was to exclude patients who diagnosed diabetes. # The sensitivity analysis was to exclude patients who diagnosed non-diabetes. HR, hazards ratio; CI, confidence interval; BMI, body mass index; KPS, karnofsky performance status; CTI, C-reactive protein-triglyceride glucose index; TSF, triceps skin fold.

**Patients who died within six months were excluded***

OS ^a^: Unadjusted.

OS ^b^: Adjusted for age, sex, BMI and TNM stage.

OS ^c^: Adjusted for age, sex, tumor stage, BMI, tumor types, KPS, surgery, chemotherapy, radiotherapy, smoking, alcohol, nutritional intervention, hypertension, coronary heart disease, and diabetes.

OS ^d^: Adjusted for age, sex, tumor stage, BMI, tumor types, KPS, surgery, chemotherapy, radiotherapy, smoking, alcohol, nutritional intervention, hypertension, coronary heart disease, diabetes, and TSF.

**Patients with diabetes were excluded^#^ and Patients with non-diabetes were excluded^&^**

OS ^a^: Unadjusted.

OS ^b^: Adjusted for age, sex, BMI and TNM stage.

OS ^c^: Adjusted for age, sex, tumor stage, BMI, tumor types, KPS, surgery, chemotherapy, radiotherapy, smoking, alcohol, nutritional intervention, hypertension, and coronary heart disease.

OS ^d^: Adjusted for age, sex, tumor stage, BMI, tumor types, KPS, surgery, chemotherapy, radiotherapy, smoking, alcohol, nutritional intervention, hypertension, coronary heart disease, and TSF.
